# Supplementary material for: Activation of mGlu2 Receptors Rescues Persistent Post-Methamphetamine Deficit in Object-in-Place Recognition Memory
Source: Brain Sci. 2026 Jun 28;16(7):682. doi: 10.3390/brainsci16070682 (PMC13406505; doi:10.3390/brainsci16070682)
Supplement: Supplementary file 1 [file brainsci-16-00682-s001.zip › brainsci-4344562-supplementary.pdf]

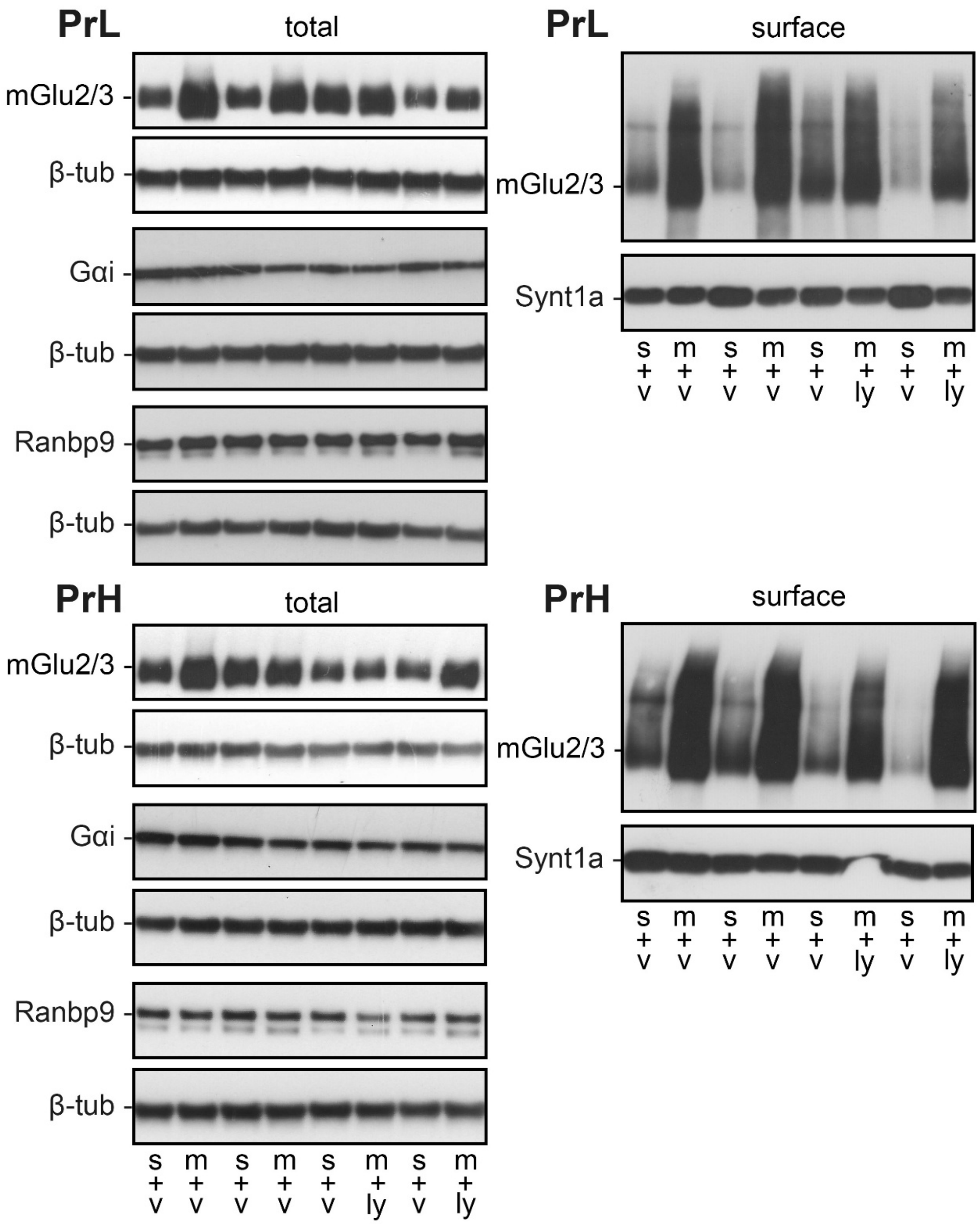

Supplemental Figure S1. Immunoblotting analysis of total and surface proteins in the PrL and PrH. (Left) Representative immunoblot signal in total tissue lysates from the PrL and PrH cortices for: mGlu2/3 receptor (dimer, ~ 250 kDa), Gαi protein (~40 kDa), RanBP9 (~90 kDa), and loading control β-tubulin (~55 kDa). (Right) Representative immunoblot signal in cell surface fraction from the PrL and PrH cortices for: mGlu2/3 receptor (dimer, ~250 kDa) and loading control syntaxin-1a (~35 kDa). The immunoreactive bands shown are representative and originate from the same sequence of rat brain samples (PrL/PrH and total/surface), loaded in the following group order: sal+veh, meth+veh, meth+LY-487379, meth+LY-487379, meth+LY-487379, meth+LY-487379. Band intensities for a given lane may not precisely match the group means shown in Fig. 5, consistent with normal sample-to-sample variability in protein analysis of heterogeneous tissues (e.g., brain). This variability does not reflect sample degradation or unequal loading, as the loading controls show a uniform signal across all lanes.
